# Handheld Active Add-On Control Unit for a Cable-Driven Flexible Endoscope

**DOI:** 10.3389/frobt.2019.00087

**Published:** 2019-09-20

**Authors:** Julie Legrand, Allan Javaux, Mouloud Ourak, Dirk Wenmakers, Tom Vercauteren, Jan Deprest, Sebastien Ourselin, Kathleen Denis, Emmanuel Vander Poorten

**Affiliations:** ^1^Laboratory of Robot-Assisted Surgery, Department of Mechanical Engineering, KU Leuven, Leuven, Belgium; ^2^Department of Imaging and Biomedical Engineering, King's College London, London, United Kingdom; ^3^Department of Development and Regeneration, KU Leuven, Leuven, Belgium

**Keywords:** add-on system, TTTS, medical robots, flexible robots, mechanism design

## Abstract

The instruments currently used by surgeons for *in utero* treatment of the twin-to-twin transfusion syndrome (TTTS) are rigid or semi-rigid. Their poor dexterity makes this surgical intervention risky and the surgeon's work very complex. This paper proposes the design, assembly and quantitative evaluation of an add-on system intended to be placed on a commercialized cable-driven flexible endoscope. The add-on system is lightweight and easily exchangeable thanks to the McKibben muscle actuators embedded in its system. The combination of the flexible endoscope and the new add-on unit results in an easy controllable flexible instrument with great potential use in TTTS treatment, and especially for regions that are hard to reach with conventional instruments. The fetoscope has a precision of 7.4% over its entire bending range and allows to decrease the maximum planar force on the body wall of 6.15% compared to the original endoscope. The add-on control system also allows a more stable and precise actuation of the endoscope flexible tip.

## 1. Introduction

The twin-to-twin transfusion syndrome (TTTS) is an ailment affecting up to 9% of all monochorionic diamniotic twin pregnancies (Lewi et al., 2010). This condition is caused by an unbalanced placental vascularization between the monochorionic twins. If left untreated, perinatal mortality rate exceeds 90% (Robyr et al., 2006). Endoscopic laser ablation (ELA), a relatively recent *in utero* treatment, has shown to lead to excellent outcomes (Chang, 2013). ELA consists in introducing an endoscope equipped with a therapeutic laser through the abdominal and uterine walls. By visualizing the placenta with the endoscope, the surgeon can laser, using the therapeutic laser, the anastomoses, i.e., the placental vessels responsible for the blood transfer between the twins (Ville et al., 1998). However, this surgery induces iatrogenic (i.e., caused by the surgery itself) preterm premature rupture of membranes (iPPROM) in about 30% of the cases (Beck et al., 2012). This risk is mainly due to the instruments used during the procedure. These instruments are rigid or semi-rigid with poor controllability, which forces the surgeons to approach the vessels under complex angles and therefore apply large forces on the uterus and the fetal membranes. This excessively exercised pressure might lead to tissue damage and/or to iPPROM.

In order to limit the forces applied at the insertion site, some dedicated endoscopes have been proposed in the literature for the targeted procedure. Yamanaka et al. (2010) created a rigid endoscope, equipped with a steerable mirror, allowing to control the focus of the laser beam. However, this endoscope only offers a limited steering angle of the laser, making it impractical in case of anterior placentas. The instrument also does not provide any steering of the camera preventing altering of the visual field of view. Moreover, the large diameter of the endoscope (7 mm) could increase the risk of preterm birth if used in fetal surgery, as suggested by Petersen et al. (2016). Petersen et al. indeed showed that a strong correlation exists between the diameter of fetoscopes and the risk of preterm birth. Improved fetoscopes for TTTS treatment therefore need to have a small external diameter (ranging between 2.3 and 4.0*mm*, similar to the already existing rigid ones) and offer a large steering angle for both the camera and the laser. Some steerable flexible manipulators have been introduced to fulfill these specifications. Harada et al. (2007) and Zhang et al. (2011) concatenate a large number of ball joints through which cables are routed. The ball joints are actuated by these cables. Yamashita et al., on the other hand, use an extra rigid link controlled in rotation in order to activate the joints of their fetoscope (Yamashitaa et al., 2006). Yao et al. (2014), however, concatenates universal joints and a prismatic joint to create a joystick used to control the distal tip of the fetoscope via cables. Even though these instruments present interesting features for TTTS treatment, among others large workspace and small external diameter, they only foresee the insertion of one tool (a laser, a camera or a forceps). Hence, an extra instrument needs to be used for illumination and/or visualization purposes. This means that extra incisions need to be performed on the body wall, potentially leading to additional complications. Also, and since these instruments are cable actuated, large amounts of frictions are expected, which can make their control quite complex (Agrawal et al., 2010).

Another interesting method that has been suggested in the literature consists in foreseeing an add-on system used to enhance the controllability of commercialized flexible endoscopes. This technique could provide the fetal surgeons a steerable fetoscope already equipped with all the required tools to perform TTTS, i.e., camera, an optical fiber and a working channel, allowing to insert a coagulation laser. Moreover, this keeps the regulatory process relatively short, reducing the path to animal and/or human trials. Some robotics systems have been introduced, like the telemanipulated robotic assistant of Zorn et al. (2018) or the robotic suturing system of Cao et al. (2019). However, these systems require to make considerable changes to the commercialized instrument or endoscope. The Avicenna Roboflex™ by Elmed (Elmed Medical System, Ankara, Turkey) (Elmed Medical Systems, 2015) remotely controls every function of any flexible ureteroscope without making any modification to it. Nonetheless, this system is massive and isolate the surgeon far from the instrument which could, in case of emergency, delay the surgeon's intervention. In response to these limitations, some handheld devices have been proposed. For example, Ruiter et al. (2012) suggested an add-on device with a joystick to be placed on a traditional endoscope for colonoscopy procedures. Fang et al. (2012) designed an add-on system actuated via two push buttons that can be mounted on a flexible rhino endoscope for Ear Nose and Throat (ENT) diagnoses. Such active devices, i.e., devices driven by actuators, could be an inspiration for the conception an active fetoscope. But these devices are not compatible with the targeted procedure. Both systems are heavy because they make use of traditional motors. Also, these instruments need to be manipulated using two hands, like current instruments. Their use for TTTS would thus require an extra surgeon in addition to the one manipulating the ultrasound scan. Latter tracks the instrument and helps to localize the fetuses in the womb.

This study proposes a modular add-on system intended to be placed on a passive (i.e., activated by the human operator only) commercialized flexible endoscope. The passive cable-driven flexible ureteroscope FLEX-X^2S^ (Karl Storz Endoskope, Tuttlingen, Germany) was chosen as the commercialized platform. The FLEX-X^2S^ is an ureteroscope that has been sometimes used by fetal surgeons in very delicate TTTS to visualize parts of the placenta that are not observable with their current instruments. The shaft of the FLEX-X^2S^ is, however, too soft, and manipulation in its current form is extremely difficult due to, among others the thickness and toughness of the abdominal wall. The flexible ureteroscope is composed of all the features required by a fetoscope, i.e., a high-quality lens, a light source, and a working channel allowing the insertion of a therapeutic laser fiber. Its steering is cable-based. The user adjusts the direction of the distal tip by operating a lever at the back of the handle. The actuation of the lever of the ureteroscope allows a 270° deflection in two directions in a single plane. The designed add-on system is lightweight and allows an active one-handed manipulation of the endoscope. A first version has been presented on a LithoVue which is a disposable ureteroscope system from Boston Scientific (Natick, USA, Legrand et al., 2018). This validated the feasibility of such a single-handed add-on system. Nonetheless, the image quality was suboptimal. Furthermore the prototype suffered from limited controllability as only an open-loop hysteresis control approach was adopted. This paper transfers the work to the FLEX-X^2S^, which offers better image quality. Moreover, a novel dedicated add-on is developed and the control is improved with a more powerful control approach. Finally, the paper provides a more detailed and quantified user-in-the-loop assessment of the instrument performance. The layout of this paper is as follows: the new design of the add-on system as well as its control system is described in section 2. In section 3, experiments are conducted in order to confirm the added value of the controller, and the adapted instrument is tested on mixed-reality simulator by novices. The experiments are discussed, conclusions are drawn and further work on the fetoscope is suggested in section 4.

## 2. Materials and Methods

### 2.1. Design and Assembly

**Figure 1:**
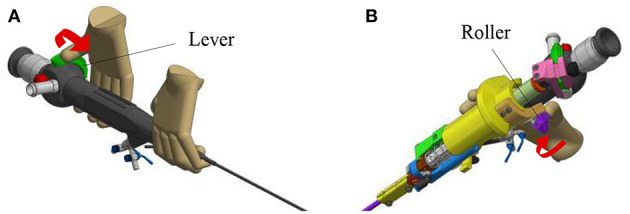
Overview of the hand pose. The red arrow represents the movement that makes the flexible tip bend: **(A)** Pose on the FLEX-X^2S^. **(B)** Pose on the new add-on system.

In order to create an active fetoscope from a passive flexible ureteroscope FLEX-X^2S^, a dedicated add-on system needs to be designed to actively actuate the handle of the ureteroscope while ensuring an ergonomic position of the hand. At the same time it should be possible to actuate the instrument single-handed. Indeed, the original ureteroscope FLEX-X^2S^ is currently actuated via a lever as shown in Figure 1A. By pulling the lever toward the distal direction (red arrow on Figure 1A), the flexible tip bends via the cable system inside the instrument. An opposite movement, i.e., pulling toward the proximal direction allows the flexible tip to relax and come back straight. The hand pose requires the use of the other hand to precisely position the instrument inside the womb. Also, the passive actuation of the ureteroscope leads to jerky and imprecise flexible tip control due to friction in the cable system present in the instrument. The user would actually need to mentally compensate for this friction, which is, in practice, quite complex. The new add-on system, however, can offer a stable and relatively precise control of the instrument flexible tip thanks to its active system. Moreover, its manipulation can be done using one single hand (Figure 1B). The user grabs the add-on frame (yellow part on Figure 1B), and with his/her thumb, easily commands the distal deflection by operating a roller. Actuating the roller clockwise allows the tip to bend, whereas its counterclockwise actuation makes it come back straight.

**Figure 2:**
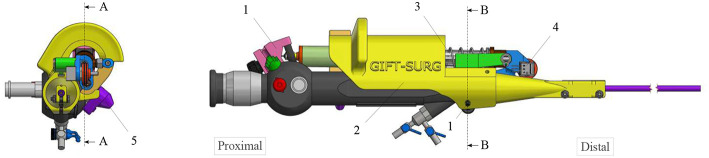
Overview of the add-on system fixed on the ureteroscope: 1. Fixations on the instrument; 2. Add-on frame; 3. Actuation unit; 4. Sensing unit; 5. Scroll wheel.

The new add-on device is designed for an easy assembly and disassembly onto the FLEX-X^2S^. This add-on is intended to be disposable. An overview of the system is presented in Figure 2. The system comprises three main parts: the add-on frame; composed of a large piece to be grasped by the hand and a roller interface to control the flexible tip of the instrument, the actuation unit; allowing the active actuation of the tip and the sensing unit; composed of a sensor allowing the proper control of the actuation unit. The operation roller (element 5 on Figure 2) is the user interface that allows to control the bending of the distal flexible tip of the fetoscope.

**Figure 3:**
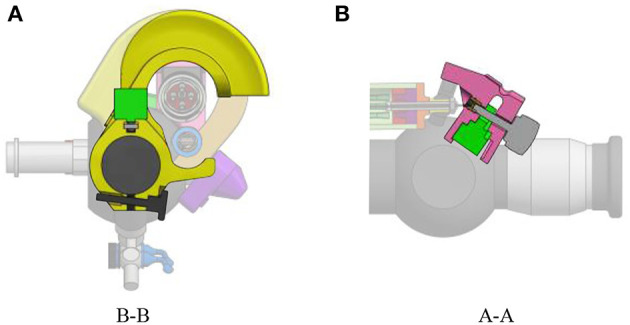
Fixation of the add-on system on the ureteroscope FLEX-X^2S^: **(A)** B-B cut section of the distal fixation, from Figure 2. **(B)** A-A cut section of the proximal fixation, from Figure 2.

The add-on system is fixed to the instrument at two different locations (Figure 2). At the proximal part of the handle, the connection is made with a flex-hinge and a screw (Figure 3B). The add-on is moved from the tip of the instrument along the longitudinal axis until it reaches the handle portion and the hinge is seated against a surface of the handle portion. Then, the screw is tightened, fixating the add-on at the proximal part of the handle. At the distal part of the handle, the 3D printed add-on frame is clamped on the ureteroscope via a screw, just as a clamp ring (Figure 3A). The clamp ring can be slid sidewards over the native lever and then fixed by screwing the fixation screw tight. The add-on system is fixed so that the lever position of the original ureteroscope corresponds, at rest, to the maximum bent configuration. The total time for clamping the add-on on the ureteroscope is about 1 min. A rigid shaft has also been added around the current soft shaft of the ureteroscope providing stability to the shaft. The current shaft of the FLEX-X^2S^ is 650 mm long. However, the shaft of the instruments used for the TTTS procedures is only about 200–400 mm (Klaritsch et al., 2009). Before taking any decision about cutting and shortening the shaft of this expensive commercialized ureteroscope, it is investigated in section 3 how suitable the adapted active fetoscope with a shortened shaft is for TTTS through use of virtual reality.

In order to keep the add-on system lightweight, McKibben muscles were chosen as actuators. McKibben muscles produce high output forces and displacements even for low diameter muscles. These actuators also achieve good positioning accuracy (De Volder et al., 2011). In the actuation unit of the add-on system, these McKibben muscles are placed in parallel. The muscles, once pressurized will pull on the lever, making the distal angle tend toward 0° (i.e., the flexible tip tends to be straighter). An antagonist spring ensures the return of the instrument tip to the rest position when the muscles are deflated. This rest position is actually the maximum bent configuration due to the design choice of the add-on system fixation onto the ureteroscope. Indeed, it is fixed so that, at rest, when the muscles are depressurized, the lever position corresponds to the maximum bent configuration of the distal tip. A maximum unidirectional bending angle, equal to 180°, was foreseen. The unidirectional feature of the distal tip was asked by fetal surgeons in order to simplify as much as possible the manipulation of the instrument (Legrand et al., 2018). This does not restrict the instrument's number degree of freedom, since the surgeon can always twist the instrument along its longitudinal axis.

**Figure 4:**
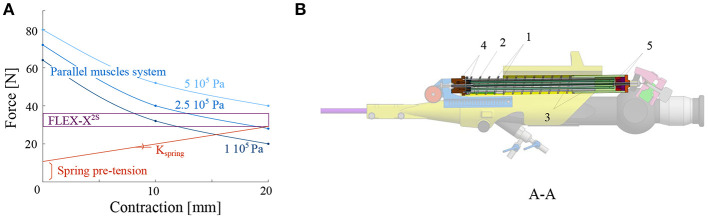
Design of the actuation unit of the add-on system: **(A)** Graph of the design principle of the actuation system; **(B)** Overview of the actuation unit: 1. McKibben muscles; 2. spring; 3. telescopic linear guide; 4. distal pressure connection; 5. proximal conical coupling.

The minimum force that needs to be applied on the lever of the original FLEX-X^2S^ in order to make the distal flexible tip bend from 0° to 180° was experimentally evaluated to be 7*N* while the lever needs to move 20 mm (represented by the purple rectangle on Figure 4A). These 7*N* represent the friction forces involved in the FLEX-X^2S^. This friction was supposed to be more or less constant during the entire contraction. The stiffness of the antagonist spring needs to be as low as possible in order to allow the muscles to easily counteract it in 20 mm of contraction, but large enough to ensure that the scope bends back to its rest position. A large spring of 17.6 mm diameter and of stiffness 1.3 N/mm was therefore chosen (represented by the red line on Figure 4A). In order to calculate the number of McKibben muscles needed to actuate the system over its entire range (0–180°), the force curve of one muscle was experimentally obtained. This curve was multiplied by an integer (i.e., the number of muscles) so that the obtained force could counterbalance the friction and the spring forces. From Figure 4A, one can derive that four muscles could suffice to counteract the combination of the FLEX-X^2S^ friction and the spring. Indeed, if the force generated by the spring is added to the friction of the Storz's ureteroscope (Figure 4A), one can conclude that four muscles are sufficient if actuated with an input pressure of approximately 5·10^5^*Pa* at 20*mm* of contraction. The output force generated by the four muscles in parallel is represented by the blue curves on Figure 4A, at different pressurization values.

Four McKibben muscles of 2 mm diameter (in relaxed state) and 150 mm length were thus placed in parallel, surrounded by the spring of 17.6*mm* diameter and 1.3*N*/*mm* stiffness (RS components, Corby, UK) (Figure 4B). In order to protect the muscles and drive the linear movement, a linear telescopic guide surrounding the muscles and inside the spring is foreseen (Figure 4B). The guide is 18*mm* outer diameter and made of iglidur® H1 (Igus, Cologne, Germany). At the distal part of the actuation unit, the muscles are closed and fixed via a conical coupling. At the proximal part of the unit, the muscles are also fixed with the same system and linked to the pressure source via a Festo elbow thread-to-tube adapter (Festo, Esslingen am Neckar, Germany). The actuation unit is fixed to the add-on frame, at the proximal end using a ball joint linked to the proximal fixation, allowing free motion of the ureteroscope lever (Figure 3B). At the distal end, the actuation unit is screwed to the sensing unit (Figure 4B).

It is important to note that the dimensioning of the actuation unit was done without taking the effect of the therapeutic laser into account. The surgeons are currently using a Nd-YAG or diode laser to perform the coagulation. But the large stiffness of these laser types makes it not suitable for use in flexible instruments. It is notably investigated whether these fibers can be replaced by very thin flexible fibers (about 500 microns outer diameter) like Holmium YAG (Ho-YAG) lasers (Brenner et al., 1997). It was experimentally verified, by inserting such a fiber in the working channel of the FLEX-X^2S^, that it has no impact on the flexible fetoscope output angle.

**Figure 5:**
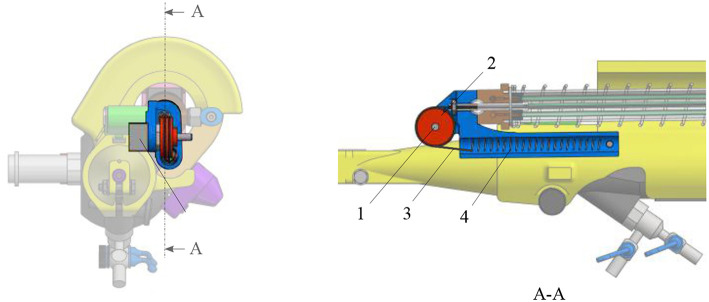
Overview of the sensing unit: 1. potentiometer; 2. pulley; 3. cable; 4. tensioning spring.

The position of the ureteroscope lever is measured using a wire coupled to a linear potentiometer 100*kΩ* (Bourns, Bedford, UK) (Figure 5). The wire is fixed at one end at the moving proximal part of the actuation unit. It passes through a pulley, and is fixed at its other end to a pre-tensioned spring with a 0.12*N*/*mm* stiffness. The pulley is directly coupled in its center to the sensing potentiometer. The tension spring is used to guarantee a minimum tension in the sensing cable, avoiding backlash during actuation.

A scroll wheel is fixed on the add-on frame. It is used as an input interface for the surgeon to control the device (Figure 2). Its placement allows an ergonomic pose of the hand (Figure 1B). The scroll wheel is directly linked to a linear potentiometer 100*kΩ* (Bourns, Bedford, UK). When the roller is activated by the surgeon, the value of the interface potentiometer will define the amount of pressure that needs to be sent to the four muscles. The muscles will then inflate and pull on the ureteroscope lever. Latter will bend the distal flexible tip of the instrument via the internal cables present inside the FLEX-X^2S^. By inflating, the four muscles also pull on the sensing cable (Figure 5). The pulley will then rotate. The value of the sensing potentiometer is used as a reference command for the control system regulating the muscle contraction in closed loop.

### 2.2. Identification and Characterization

**Figure 6:**
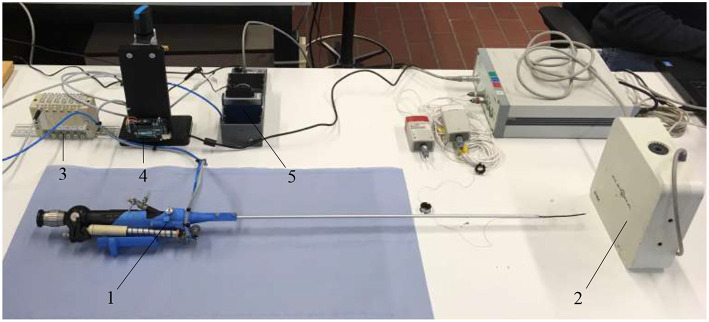
Experimental setup used to characterize the adapted FLEX-X^2S^: 1. Adapted FLEX-X^2S^; 2. Aurora system; 3. Proportional valves; 4. Arduino; 5. Ethercat.

In order to optimize the controllability of the adapted FLEX-X^2S^, i.e., that the surgeon will feel a linearity between the interfacing scroll wheel and the actual bending angle of the fetoscope, the behavior of the instrument was characterized. To do so, an experimental set-up has been developed (Figure 6). The air pressure is regulated via an electrovalve ITV0050-3MN-Q (SMC, Tokyo, Japan) to the McKibben muscles. OROCOS, a middleware for real time robots control as well as an EtherCat module NI9144 (National Instrument, Texas, USA) and AO DAQ NI9263 (National Instrument, Texas, USA), are used to control the electro-valve. The latter is supplied by a Cyclon 215 air compressor (CompAir, Wisconsin, USA). The output of the pneumatic valve is linked to the four McKibben muscles inside the adapted instrument. The proportional valve has a response time of 0.1 s when no load is applied, and is supplied with a maximum pressure of 0.15 MPa. The corresponding bending angle of the flexible tip is computed by tracking two Aurora encapsulated 5DOF electromagnetic sensors 0.9 x 6 mm (Aurora-NDI Medical, Cleveland, USA). One sensor was placed on the rigid shaft of the instrument. The second one was placed at the very end of the distal bendable tip, inside the working channel. By tracking both sensor orientations with the Aurora field generator (Aurora-NDI Medical, Cleveland, USA), the bending angle of the instrument can be calculated. The exact position of the sensing potentiometer inside the instrument is also recorded via an Arduino. Finally, a pressure sensor (Keller PA-21Y, Winterthur, Switzerland) was added at the output of the valves. OROCOS is used to acquire the data and achieve the control.

**Figure 7:**
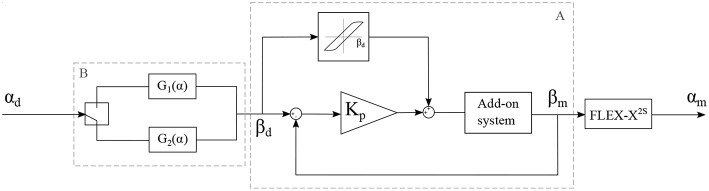
Control schema of the adapted instrument, with α_*d*_: the desired bending angle of the distal tip, α_*m*_: the measured bending angle of the distal tip, β_*d*_: the desired lever angle and β_*m*_: the measured lever angle.

**Figure 8:**
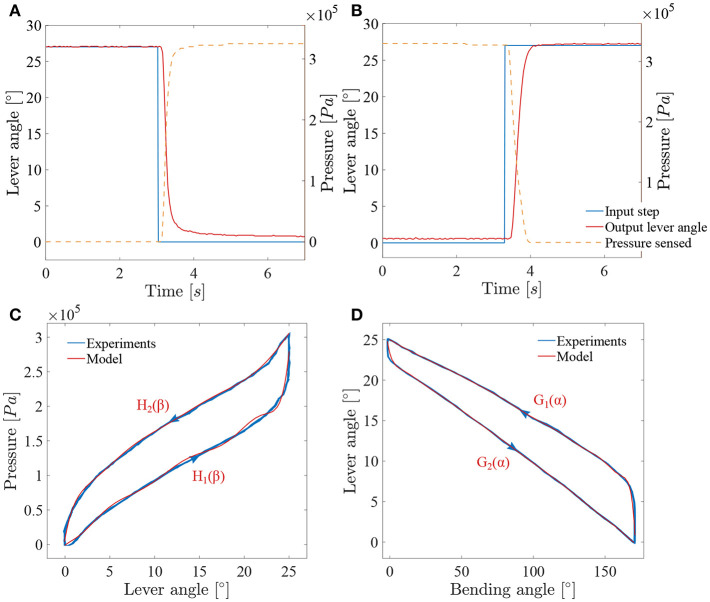
Results of the characterization of the adapted ureteroscope: **(A)** System response to a decreasing lever angle step (loading); **(B)** system response to an increasing lever angle step (unloading); **(C)** muscles hysteresis; **(D)** FLEX-X^2S^ friction hysteresis between the distal bending angle and the lever angle.

The add-on system with McKibben muscles is controlled in closed loop, using the potentiometer situated in the sensing unit as a sensor for the position of the lever. The FLEX-X^2S^ is, however, controlled in open-loop based on the instrument characterization. The muscles in the add-on system introduce an important pressure-to-contraction hysteresis. Even if a sensor is already used to control the muscle contraction in closed loop, adding a hysteresis compensation to it can help the system converge to the correct muscle contraction value faster. Therefore, the closed loop governing the desired muscle contraction and therefore the desired ureteroscope lever angle β_*d*_ is a classical closed loop with an extra hysteresis compensation added to the output of the controller, just before entering the system (see part A in Figure 7). The pressure-to-contraction hysteresis is experimentally obtained (Figure 8C). In order to extend the control to the distal tip of the instrument, a relation needs to be established between the distal bending angle α and the lever angle β. Due to the small dimensions of the ureteroscope distal tip, it is not possible to insert an extra sensor at the tip of the instrument, or anywhere between the add-on and the distal tip. Therefore, this part will be controlled using an open-loop based on the instrument characterization. The relation between the desired distal bending angle α_*d*_ and the desired lever angle β_*d*_ presents an important hysteretic behavior due to the cable friction inside the uretereoscope (Figure 8D). This friction hysteresis will therefore be compensated by approximating two functions governing this hysteresis, one for the loading curve and one for the unloading curve (see part A in Figure 7). The whole control system of the fetoscope therefore forms a feed-forward control regulating the distal bending angle.

In order to characterize the closed loop system, two pressure steps from respectively 0*Pa* to 3.3·10^5^*Pa* and 3.3·10^5^*Pa* to 0*Pa* were applied to the system and the lever angles were measured (see Figures 8A,B). The sensing potentiometer values were recorded and mapped to the lever angle range. These steps correspond respectively to a bending angle from 170° to 0° and from 0° to 170° of the distal tip. The add-on system dynamics can be seen as a first order system with a loading delay θ_*load*_ = 0.095*s*, and a loading time constant τ_*load*_ = 0.16*s*. Unloading is somewhat slower with an unloading delay θ_*unload*_ = 0.18*s*, and an unloading time constant τ_*unload*_ = 0.23*s*. These time values were determined based on a single cycle. Under loading, the system does not completely reach the targeted lever angle. The error value is evaluated to 0.8° and is due to the friction inside the ureteroscope. A proportional gain *K*_*p*_ is therefore used in this closed-loop controller multiplying the position error to obtain a steering pressure command.

The compensation of the muscle hysteresis will be added to this error (Figure 7). The muscle hysteresis has been measured on 15 pressure cycles using a triangle wave going from 0*Pa* to 3.3·10^5^*Pa* and back (see Figure 8C). The repeatability of the muscle hysteresis measurement has been evaluated to be 6.3% of the entire lever angle range. In order to create the hysteresis compensation, the loading and unloading curves of the muscle hysteresis (Figure 8C) were modeled using a ninth order polynomial:

$$\begin{array}{l} H_{1}(\beta_{d})=1.3\cdot 10^{-9}\beta_{d}^{9}-1.3\cdot 10^{-7}\beta_{d}^{8}+5.5\cdot 10^{-6}\beta_{d}^{7}\\ \;-1.2\cdot 10^{-4}\beta_{d}^{6}+1.\;6\cdot 10^{-3}\beta_{d}^{5}\\ \;-1.1\cdot 10^{-2}\beta_{d}^{4}+4\cdot 10^{-2}\beta_{d}^{3}-5.6\cdot 10^{-2}\beta_{d}^{2}\\ \;+0.1\beta_{d}-1\cdot 10^{-2}\\ H_{2}(\beta_{d})=3.3\cdot 10^{-10}\beta_{d}^{9}-4.2\cdot 10^{-8}\beta_{d}^{8}+2.3\cdot 10^{-6}\beta_{d}^{7}\\ \;-6.7\cdot 10^{-5}\beta_{d}^{6}+1.2\cdot 10^{-3}\beta_{d}^{5}\\ \;-1.3\cdot 10^{-2}\beta_{d}^{4}+8.3\cdot 10^{-2}\beta_{d}^{3}-0.3\beta_{d}^{2}+0.7\beta_{d}\\ \;+8.6\cdot 10^{-2} \end{array}$$

with β_*d*_, the desired lever angle in degrees. The Root-Mean-Square Error (RMSE) value characterizing the model has been evaluated to be 8.58·10^3^*Pa* for the loading curve and to 3.8·10^3^*Pa* for the unloading curve. Therefore, a different proportional gain *K*_*p*_ will be defined for the loading (named *K*_*pload*_) and unloading curve (*K*_*punload*_). This is logical as the mechanical components that are actually creating the bending of the instrument differs for loading and unloading. During the loading, the McKibben muscles are working against the spring whereas for the unloading, only the spring is active and ensures the return to the rest position.

However, the instrument end-effector is not uniquely determined for a given lever pose. Friction between cable and cable-routing also leads to a hysteretic behavior between the distal bending angle and the lever. Therefore, and in order to control the distal bending angle, the function relating the lever angle to the distal bending angle has been characterized in the same manner as for the muscle hysteresis over 15 cycles (Figure 8D). This function represents an asymmetric hysteresis, which is due to the cable friction inside the FLEX-X^2S^ and can be approximate by two curves, *G*_1_(α) for the loading phase and *G*_2_(α) for the unloading phase:

$$\begin{array}{l} G_{1}(\alpha )=-2.2\cdot 10^{-27}\alpha^{15}+2.2\cdot 10^{-24}\alpha^{14}-9\cdot 10^{-22}\alpha^{13}\\ \;+2\cdot 10^{-19}\alpha^{12}-2.1\cdot 10^{-17}\alpha^{11}\\ \;-1.9\cdot 10^{-16}\alpha^{10}+3.8\cdot 10^{-13}\alpha^{9}-5.8\cdot 10^{-11}\alpha^{8}\\ \;+4.8\cdot 10^{-9}\alpha^{7}-2.5\cdot 10^{-7}\alpha^{6}\\ \;+8.5\cdot 10^{-6}\alpha^{5}-1.8\cdot 10^{-4}\alpha^{4}+2.3\cdot 10^{-3}\alpha^{3}\\ \;-1.5\cdot 10^{-2}\alpha^{2}-0.1\alpha +24.9\\ G_{2}(\alpha )=-1.1\cdot 10^{-27}\alpha^{15}+1.5\cdot 10^{-24}\alpha^{14}-9.4\cdot 10^{-22}\alpha^{13}\\ \;+3.5\cdot 10^{-19}\alpha^{12}-9\cdot 10^{-17}\alpha^{11}\\ \;+1.6\cdot 10^{-14}\alpha^{10}-2.1\cdot 10^{-12}\alpha^{9}+2\cdot 10^{-10}\alpha^{8}\\ \;-1.4\cdot 10^{-8}\alpha^{7}+7.2\cdot 10^{-7}\alpha^{6}\\ \;-2.6\cdot 10^{-5}\alpha^{5}+6.7\cdot 10^{-4}\alpha^{4}-1.1\cdot 10^{-2}\alpha^{3}\\ \;+0.1\alpha^{2}-0.7\alpha +23.3 \end{array}$$

with α, the bending angle of the distal tip in degree. The RMSE value characterizing the model with respect to the characterization is 1.02° for the loading curve and 0.28° for the unloading curve. This open loop control part can be added to the control schema in order to extend the control to the distal bending angle. The overall control scheme is depicted in Figure 7.

### 2.3. Instrument Evaluation

**Figure 9:**
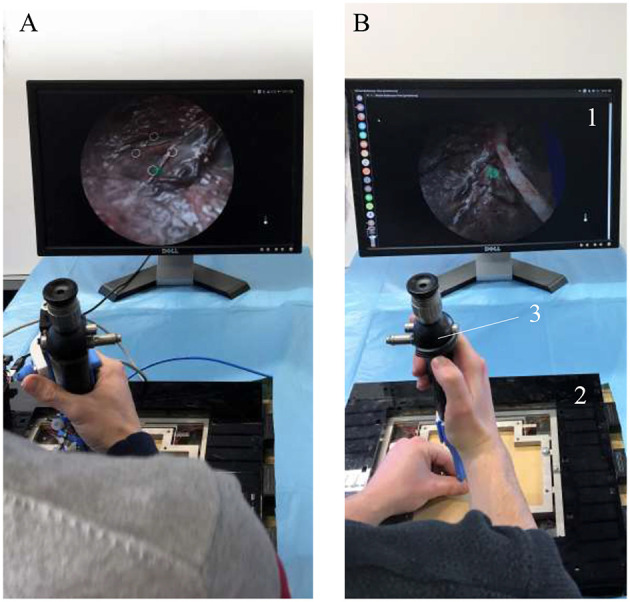
Overview of the mixed-reality simulator during experiments: 1. Screen; 2. Force sensor; 3. Instrument; **(A)** Experiment using the active flexible fetoscope. **(B)** Experiment using the FLEX-X^2S^.

The developed active flexible fetoscope was tested using a mixed-reality simulator described and validated by Javaux et al. (2018), which simulates a realistic TTTS surgery. The system benefits from the usage of both physical and virtual components. The user inserts a surgical instrument of his choice through a 4 cm thick-synthetic body wall phantom ensuring realistic haptic during manipulation. The inside of the womb is rendered by a Virtual Reality (VR) system, which provides the user a virtual scope view of the environment. By the means of a foot pedal, the user is able to activate the virtually equipped laser and thus coagulate the anastomoses on a fictive pathologic placenta. Figure 9 depicts the simulator and its different components. The screen allows the user to visualize the virtually simulated internal view of the womb. A 6 DOF force sensor is placed on the body wall in order to measure the interaction forces between the manipulated instrument and the body wall, i.e., the several layers of muscle, fat and skin separating the womb from the outside world. Two Aurora encapsulated 6DOF electromagnetic sensors (Aurora-NDI Medical, Cleveland, USA) are used to track the bending angle and the tip position and orientation of the instrument. One is placed on the rigid shaft. The second one is placed at the distal bendable tip. By tracking the position and orientation of the sensor placed at the tip, the system can provide the user a virtual view of the womb as it was coming from the integrated camera. The use of these sensors may also allow a virtual shortening of the shaft instrument. The bending tip was virtually mapped 300mm higher toward the proximal part of the instrument.

A typical experiment consists first of inserting a cannula through the synthetic body wall, then introducing the instrument inside the cannula and then beginning the required virtual task.

Five novices, without surgical expertise, were asked to conduct the following experiment using first the developed active flexible fetoscope and then the FLEX-X^2S^ with an added rigid shaft in order to stiffen its original shaft and make it usable for TTTS treatment (Figure 9). The experiment consists in accomplishing basic fetoscopic tasks, i.e., scope manipulation, selective lasering and line lasering. The users must first find the location of the four preselected anastomoses placed in diamond shape on the placenta like depicted on the screen of Figure 9A. Once found, he/she has to orient the scope as perpendicular as possible to the placenta, and laser selectively each anastomoses, seen as targets. Finally, the user is asked to connect the opposite targets by a continuous ablation line, forming in the end a cross. This experiment is repeated 12 times for one instrument, making the user ablate 48 anastomoses and 24 lines. For each novice, only the six final crosses were taken into account in the result analysis. The six previous crosses were considered as training to get used to the virtual environment as well as to the instrument.

## 3. Results

The control algorithm of the adapted ureterosope was first tested to check whether the performance is acceptable for use as a handheld instrument. Then, a group of novices were invited to test the instrument in Virtual Reality (VR) by means of a mixed-reality surgical trainer (Javaux et al., 2018). The real instrument was used and tracked and a virtual environment was updated accordingly. During the last experiment, two important questions were investigated on the basis of some defined quantitative metrics: “Can the fetoscope (i.e., Storz's ureteroscope equipped with the novel add-on and a virtually shorten shaft) be used for a TTTS procedure?,” and “What is its added-value in comparison with the standard Storz's ureteroscope?”

### 3.1. Verification of Controllability

In order to test the performance of the control system, a series of sine wave input angles were applied to the instrument. Each sine wave has an amplitude equal to half of the maximum angle range of the instrument. For all the controllability tests, the *Kpload* was fixed to 1 and the unloading *Kpunload* to 3. A sine wave frequency of 0.01*Hz* was first applied to check the added value of respectively the muscle hysteresis compensation and the friction hysteresis compensation.

**Figure 10:**
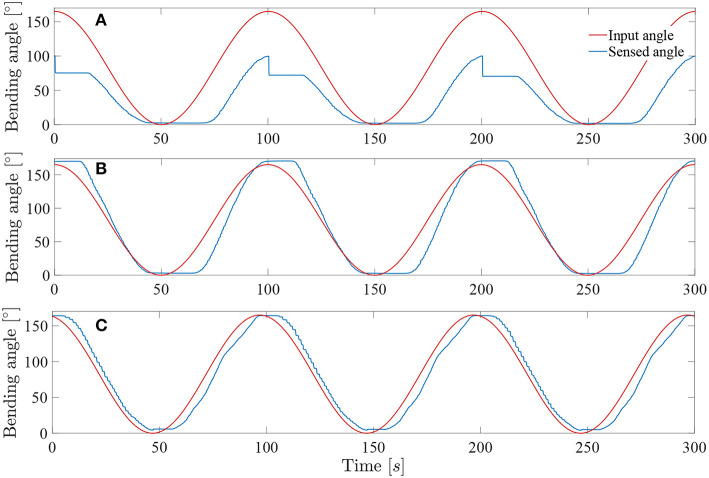
Response of the system to a 0.01*Hz* sine wave angle with *Kpload* = 1 and *Kpunload* = 3: **(A)** Simple closed loop system with linear assumption between α and β. **(B)** Closed loop system with muscle hysteresis compensation. **(C)** Closed loop system with muscle hysteresis compensation and friction compensation.

Figure 10A depicts the angle response of the instrument to a 0.01*Hz* sine with an amplitude equal to half of the instrument full range when a simple closed loop is used (i.e., with no muscle or friction hysteresis compensation). An important drop in angle is visible when the input angle reaches a maximum. This is due to the fact that two different proportional gains were chosen for the loading and the unloading curve. The mean distal angle error is evaluated to be 47.7° for this simple controller over 300*s* of test. The Figure 10B depicts the angle response to the same input sine when a closed loop is used with only muscle hysteresis compensation (i.e., with the block A on Figure 7 only). The instrument can now reach a full-angle range compared to the previous case, but still presents an important position error due to the cable friction inside the uretereoscope. This mean error was evaluated to be 15.8°, which is three times better than with the simple control loop. Finally, the same test was executed on the complete control loop, with muscle and friction hysteresis compensation (Figure 10C) (i.e., with both block A and B on Figure 7). The error further decrease in comparison to the controller that only uses the muscle hysteresis compensation. The mean error was evaluated to be 12.5° and the maximum error 26.3° over 300*s* of test. This persistent error is mainly due to friction inside the FLEX-X^2S^ that was not fully compensated by the implemented friction compensation. The bending angle errors evaluated throughout the different tests are reported in detail in Table 1.

**Table 1:** Summary of the bending angle errors evaluated during the controllability test for different controller: MH, muscle hysteresis compensation; MH & FH, muscle and friction hysteresis compensation.

**Controller type**	**Frequency [Hz]**	**Error mean [°]**	**Error std [°]**	**Max error [°]**
Simple controller	0.01	47.7	29.4	94.9
Controller with MH	0.01	15.8	14.2	47.5
Controller with MH & FH	0.01	12.5	6.5	26.3
	0.02	12.7	7	27.6
	0.05	12.9	7.4	28.4

**Figure 11:**
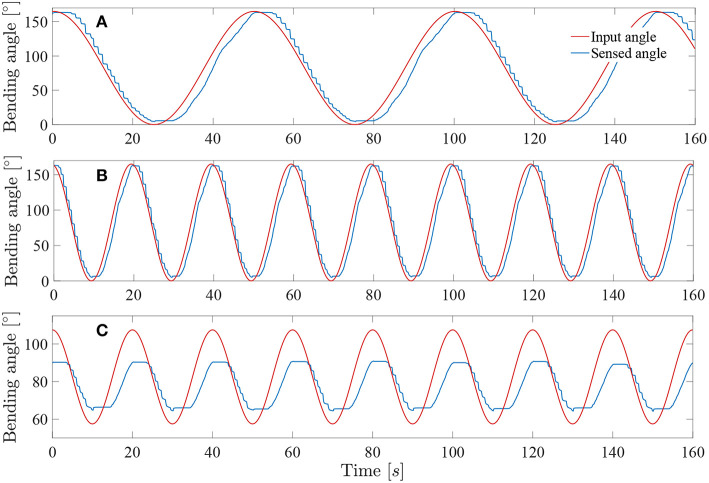
Response of the system sine wave angle with *Kpload* = 1 and *Kpunload* = 3: **(A)** Test at 0.02*Hz* on a full-angle range. **(B)** Test at 0.05*Hz* on a full-angle range. **(C)** Test at 0.05*Hz* on a 50° angle range.

The system was tested at higher frequencies: 0.02 and 0.05*Hz*. This last frequency represents approximately the actual rate of the instrument when in a realistic use. Indeed, the surgeon needs to precisely coagulate vessels on the placenta with a limited field of view available. Its movements will therefore be relatively slow for safety purposes. The result of these tests on the complete controller (i.e., with muscle and friction hysteresis compensation) are visible on Figures 11A,B. By increasing the frequency, the mean error stays approximately the same over a test period of 300*s* for both 0.02 and 0.05*Hz* angle input sinuses in comparison with the test realized with the same controller but at a frequency of 0.01*Hz*. The detailed evaluated errors for both tests are reported in the Table 1. A last test was conducted by sending a 0.05*Hz* sinus with an amplitude of 25° in the middle of the bending angle range, i.e., 85° (Figure 11C). This test allows to check if the control works also fine inside the bounds of the instrument bending angle. The sensed angle presents an average error of 10°. This is mainly because the characterization of the instrument was done on its full bending angle range. In order to solve this problem, both friction and muscles hysteresis could be characterized in greater detail, by e.g., characterizing the hysteresis over different bending angle ranges. Another solution could make use of hysteresis compensation algorithms for the muscles, like suggested by Mei et al. (2017) or Capace et al. (2019). Even though this 10° error is important, one should realize that this is a human-in-the-loop system. It is sufficient that the user feels more or less a linear relation between the input command (through the scroll wheel) and the actual output, the distal bending angle. The remaining discrepancy will be dealt by the human that closes the overall control loop. In the next section, it is investigated by user experiments if the control quality is good enough to be able to perform a TTTS procedure.

It is important to note that the linear loading and unloading gains *Kpload* and *Kpunload* were optimized for instrument frequencies between 0.01 and 0.05*Hz*, i.e., frequencies where the instrument will most likely be used for the TTTS procedure. Since the whole instrument system is highly non-linear, other gains could be searched if users want to operate at higher frequencies.

### 3.2. User Experiments

**Figure 12:**
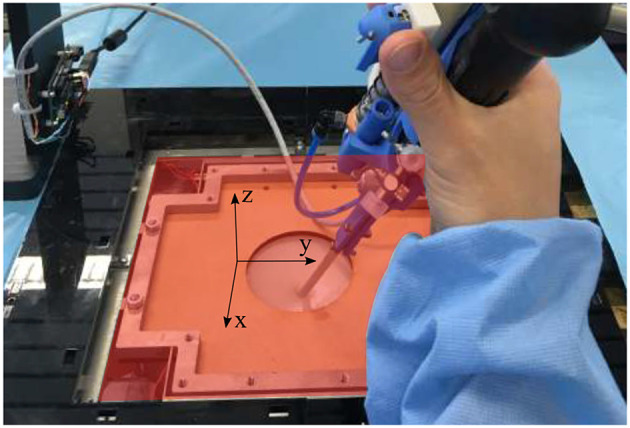
Overview of the force sensing platform and the body wall of the mixed reality simulator. The red x-y plan represents the planar plan in which the planar forces are measured, whereas the vertical forces are the forces directed along the z-axis.

During the mixed-reality test described in section 2, seven metrics were measured (see Table 2). These metrics were recorded for each experiments which are consisting in three phases: (1) An exploration phase, consisting in finding the preselected anastomoses on the placenta; (2) An adequate positioning of the tip and a first lasering phase, where the user has to orient the instrument tip perpendicular to the placenta and laser each of the four anastomoses selectively; (3) A second lasering phase, where the user has to connect the opposite anastomoses by continuous ablation lines. Some metrics have been measured during the second and third phases. The vertical and planar maximum forces were registered for both instruments, as well as the integral of the vertical and planar forces. The vertical forces are the forces in the z-direction in Figure 12, whereas the planar forces are the forces in the x-y plane in Figure 12. The integral of the forces is a measure of high forces and their duration. The derivative of the bending angle integral, the fourth used metric, measures how often and how much the instrument flexible tip was actuated during the experiments. The fifth metric is a measure of coagulation accuracy. Specifically, it evaluates a correlation coefficient between and ideal coagulation of a cross and the user performance. Finally, as last metric, the time taken to perform the second and third phases for each of the last 6 crosses was recorded. For each metric, a significance test (e.g., Wilcoxon signed-rank test) was conducted using MATLAB in order to highlight which metric presents a significant difference between both instruments. Since the test sample is small (i.e., five novices), the median and Interquartile Range (IQR) were calculated for the seven metrics and for both instruments. The metrics values are reported in Table 2. Each reported value represents a metric result for the completion of one experiment consisting in the second and third above-mentioned phases. Indeed, during the first exploration phase, large movements need to be performed to inspect the placenta and find anastomoses. Since the flexible tip of the instrument only allows small and precise displacements and orientation of tip, this feature is not used in the first phase. Since the interesting features of the active fetoscope (i.e., its flexibility and controller) are not exploited, this first exploration phase was not recorded.

**Table 2:** Comparison of the user experiments result between the FLEX-X^2S^ and the Active fetoscope.

**Metrics**	***p^a^***	**FLEX-X^2S^**		**Active fetoscope**	
		**Median**	**IQR**	**Median**	**IQR**
Vertical force maximum	** <0.001**	20.65	2.18	27.74	7.09
Vertical force integral	** <0.001**	762.65	657.20	1345.62	1185.70
Planar force maximum	**0.014**	3.90	1.77	3.66	1.86
Coagulation accuracy	0.064	0.75	0.08	0.71	0.13
Time	0.096	120.50	70.00	107.99	47.00
Flexible tip actuation	0.104	43.91	43.30	33.72	31.99
Planar force integral	0.391	68.42	56.00	84.52	73.55

a Wilcoxon signed-rank test with significance p < 0.05.

*The bold values are representing the data that are significant and passed the test with significance p < 0.05*.

## 4. Discussion

From the user experiments, and specifically from the Table 2, it can be seen than three metrics present a significant difference between the FLEX-X^2S^ and the active fetoscope. Both vertical force maximum and vertical force integral are significantly different with a much larger vertical force maximum and integral for the active fetoscope. This is obviously logical since an add-on frame has been fixed to the original ureteroscope. Even though the add-on is lightweight, it leads to a mass increase. This force could, however, be lowered if the surgeon compensates for the increased mass acting vertically. The third metric that presents a significant difference is the maximum planar force. The use of the active fetoscope can significantly decrease the maximum planar force with 6.15%. With regard to the coagulation accuracy, there is no significant difference between both instruments. Nonetheless, the coagulation accuracy is a bit better when using the FLEX-X^2S^. This result can, however, be moderated if looking at the time taken to perform the coagulation of a cross. The test subjects took indeed more time with the FLEX-X^2S^ than with the active fetoscope, which could explain the slight difference in accuracy. If no significant difference was noticed in how often and how much the flexible tip was actuated, it can yet be seen that the IQR of this metric is quite high for both instruments. This means that some users used the flexible feature during perpendicular placement of the tip with respect to the placenta and coagulation, while others used it much less. The actuation of such a flexible instrument is indeed not straightforward and requires a relatively long learning curve before being able to optimally use the advantage of the instrument flexibility. The median of the flexible tip actuation metric for both instruments reflects that the distal tip was more actuated for the FLEX-X^2S^ than for the active fetoscope. Since no significant difference can be seen in the coagulation accuracy or the time but that the planar force maximum is reduced with the active fetoscope, these results show that the actuation of the flexible tip is more stable with active fetoscope than with the FLEX-X^2S^. While the user struggled a bit more finding the desired bending angle to operate, the controller of the active fetoscope could provide a more stable and precise actuation. Finally, the planar force integral did not provide any significant difference.

By use of virtual reality to evaluate the developed active fetoscope with regard to the original FLEX-X^2S^, it could be shown that the controller of the active fetoscope was beneficial for actuation stability by looking at the flexible tip actuation metric in Table 2. Moreover, the use of the add-on system could significantly decrease the maximum planar force. This means that the iPPROM risk could be lowered if this instrument was used in a real TTTS procedure. However, the current basic hysteresis modeling approach should be improved by e.g., using hysteresis compensation (Mei et al., 2017; Capace et al., 2019) or deep learning algorithms (Li et al., 2019). This would probably encourage the operator to use the flexible feature of the instrument even more, since it would be more responsive to the user input. By making optimal use of the instrument flexible tip, the planar forces should be further reduced and the total operation time could be lowered. Another limitation of this study is that it only involves novices. The test results might differ from the novice's ones if proceeding to experts experiments. Due to scarcity of TTTS experts (FoundationTTTS, 1997-2019), the prototype should already be advanced and properly assessed in order to optimally use the surgeon's time. These assessments were the purpose of this study. Further investigation therefore needs to involve experts in order to quantitatively determine the added value of the active flexible instrument compared to the currently used rigid ones. From the preliminary experiments conducted here, we nevertheless already expect that, due to its flexible nature, the planar force could be reduced with the active fetoscope.

## Data Availability

The datasets generated for this study are available on request to the corresponding author.

## Author Contributions

JL: wrote the paper, supervised the design, implemented the control, and conducted the experiments. AJ: conducted the user experiments. MO: implemented the control, supervised the design, and experiments. DW: designed the instrument. TV, JD, and SO: provided materials and intellectual support. KD and EV: supervised the whole project.

### Conflict of Interest Statement

The authors declare that the research was conducted in the absence of any commercial or financial relationships that could be construed as a potential conflict of interest.
